# Molecular Aspects Involved in the Mechanisms of *Bothrops jararaca* Venom-Induced Hyperalgesia: Participation of NK1 Receptor and Glial Cells

**DOI:** 10.3390/toxins16040187

**Published:** 2024-04-10

**Authors:** Ariela de Oliveira Pedro Bom, Monique Dias-Soares, Raíssa Cristina Darroz Corrêa, Camila Lima Neves, Natalia Gabriele Hosch, Gabriela Gomes de Lucena, Camilla Garcia Oliveira, Rosana Lima Pagano, Marucia Chacur, Renata Giorgi

**Affiliations:** 1 Laboratory of Pathophysiology, Butantan Institute, São Paulo 05503-900, SP, Brazil; ariela.bom.esib@esib.butantan.gov.br (A.d.O.P.B.); moniquesoares@usp.br (M.D.-S.); raissa.correa@esib.butantan.gov.br (R.C.D.C.); camila.neves@fundacaobutantan.org.br (C.L.N.); gabriela.lucena@ceacleste.spdm.org.br (G.G.d.L.); 2 Postgraduate Program in Toxinology, Butantan Institute, São Paulo 05503-900, SP, Brazil; 3 Laboratory of Pain and Signaling, Butantan Institute, São Paulo 05503-900, SP, Brazil; natalia.hosh.esib@esib.butantan.gov.br; 4 Laboratory of Functional Neuroanatomy of Pain, Institute of Biomedical Sciences, University of Sao Paulo, São Paulo 05508-900, SP, Brazil; camillagarcia@alumni.usp.br (C.G.O.); chacurm@icb.usp.br (M.C.); 5 Laboratory of Neuroscience, Hospital Sírio-Libanês, São Paulo 01308-060, SP, Brazil; rosana.lpagano@hsl.org.br

**Keywords:** *Bothrops jararaca*, hyperalgesia, nervous systems

## Abstract

Accidents caused by *Bothrops jararaca* (Bj) snakes result in several local and systemic manifestations, with pain being a fundamental characteristic. The inflammatory process responsible for hyperalgesia induced by Bj venom (Bjv) has been studied; however, the specific roles played by the peripheral and central nervous systems in this phenomenon remain unclear. To clarify this, we induced hyperalgesia in rats using Bjv and collected tissues from dorsal root ganglia (DRGs) and spinal cord (SC) at 2 and 4 h post-induction. Samples were labeled for Iba-1 (macrophage and microglia), GFAP (satellite cells and astrocytes), EGR1 (neurons), and NK1 receptors. Additionally, we investigated the impact of minocycline, an inhibitor of microglia, and GR82334 antagonist on Bjv-induced hyperalgesia. Our findings reveal an increase in Iba1 in DRG at 2 h and EGR1 at 4 h. In the SC, markers for microglia, astrocytes, neurons, and NK1 receptors exhibited increased expression after 2 h, with EGR1 continuing to rise at 4 h. Minocycline and GR82334 inhibited venom-induced hyperalgesia, highlighting the crucial roles of microglia and NK1 receptors in this phenomenon. Our results suggest that the hyperalgesic effects of Bjv involve the participation of microglial and astrocytic cells, in addition to the activation of NK1 receptors.

## 1. Introduction

Snakebite envenomation represents a significant global health problem, especially in regions such as Asia, Africa and Latin America. In fact, it does so to the extent that, in 2017, the World Health Organization included it in its category A of neglected tropical diseases [1,2]. In the Americas, the majority of human snakebite envenomation is attributed to the *Bothrops* genus [1]. In Brazil, snakes of this genus are responsible for 80% of envenomation in humans, and in 2022, 19,044 snakebites caused by *Bothrops* snakes were reported [3,4]. The bite of the *Bothrops jararaca* snake, a species that is within this genus and is distributed throughout the Brazilian territory, induces local manifestations, such as pain and edema, and the injuries resulting from the inflammatory process can progress to muscle degradation, necrosis and secondary infections that can lead to loss of function or amputation of the affected limb [5]. The systemic symptoms include hemorrhages, hypotension and risk of renal failure [1,3].

The hyperalgesia induced by *Bothrops* genus snake venoms, including *Bothrops jararaca* venom (Bjv), is characterized by an increased sensitivity to pain, which can be distressing and challenging to manage effectively, given the limited efficacy of antivenom therapy in controlling local reactions resulting from envenomation [1,5,6]. Thus far, there have not been any clinically proven additional treatments to antivenom therapy that can effectively control local reactions. Some preclinical studies have shown the benefits of photobiomodulatory therapy or plant extracts in treating pain and swelling after injection of *Bothrops* venoms [7]. Deepening the understanding of the pathophysiology of pain following the bite, a fundamental characteristic of envenomation, is essential for the development of targeted and effective therapeutic interventions, as there is a clear need to explore alternative and complementary treatments for this symptom.

The pathophysiological processes involved in Bjv-induced hyperalgesia are due to the direct action of substances present in the venom on the organism, together with the release of inflammatory chemical mediators by immune cells, mainly neutrophils and macrophages, which consequently trigger sensitization of peripheral and central neurons [1,5,8].

Thus, several studies have been carried out to demonstrate which components of the *Bothrops* venom are related to the induction of hyperalgesia, as well as the inflammatory mediators involved in this process [1,5,8,9,10,11,12,13,14,15,16,17,18]. However, there is limited understanding regarding the contribution of the peripheral and central nervous systems (PNS and CNS) in the mechanisms involved in mediating this phenomenon. In relation to the central nervous system, some studies have been conducted with toxins isolated from *Bothrops* venom. In this context, microglia and astrocytes present in the spinal cord contribute to the development of the hyperalgesia that results from subcutaneous administration of Lys49 and Asp49 phospholipase A_2_ (sPLA_2_), two toxins obtained from *Bothrops asper* venom [19]. Furthermore, jararhagin, a metalloprotease isolated from Bjv, induces hyperalgesia through mechanisms that involve the participation of microglial and astrocytic cells in the process of spinal neuroinflammation [12].

Microglia and astrocytes, cells that constitute the neuroglia of the central nervous system, have been found to play crucial roles in pain processing. In models of peripheral inflammatory pain, the sensitization of spinal neurons and the activation of these glial cells in the spinal cord have been observed, phenomena that contribute to the generation of hyperalgesia [20,21,22,23]. Moreover, satellite glial cells present in the sensory ganglia of the peripheral nervous system interfere with neuronal excitability, facilitating the transmission of pain after inflammatory stimuli [23,24].

A study conducted on the mechanisms involved in local hemorrhage following intraplantar injection of Bjv suggests that neurogenic inflammation is related to this process [25]. A critical mediator of neurogenic inflammation is substance P (SP), which acts predominantly on tachykinin NK1 receptors, and is a significant component in the pathophysiology of inflammatory and painful processes [26,27,28]. The NK1 receptors (NK1Rs) are found in various regions of the nervous system, including the brain and spinal cord, and are expressed in afferent neurons as well as in non-neuronal cells such as microglia and astrocytes [29]. In peripheral tissues, NK1Rs are expressed in skin, joints and viscera, by endothelial cells, lymphocytes, monocytes and macrophages [29,30]. NK1Rs have garnered significant attention in research on pain transmission and modulation mechanisms due to their crucial role in mediating pain, particularly inflammatory pain [29,31]. However, the involvement of the NK1Rs in hyperalgesia induced by Bjv is not yet known.

In this study, our aim is to deepen the understanding of the mechanisms mediated by the peripheral and central nervous systems in the pathophysiology of Bjv-induced hyperalgesia. We employ a combination of behavioral tests, pharmacological treatments and Western blotting assays to investigate the contribution of microglia, astrocytes, satellite cells, neurons and NK1 receptors in Bjv-induced hyperalgesia, with the ultimate goal of identifying novel therapeutic strategies with which to manage the pain associated with Bjv envenomation.

## 2. Results

### 2.1. Hyperalgesia Induced by Bothrops jararaca Venom (Bjv)

**Figure 1:**
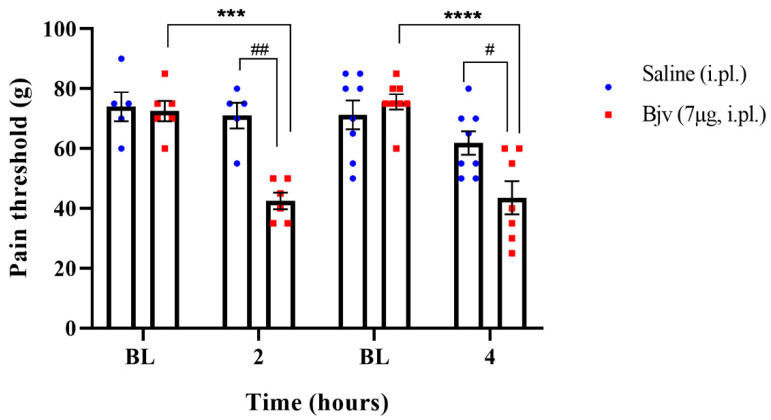
Nociceptive threshold represented in grams (g) of rats evaluated by the paw pressure test before (baseline (BL)) and two or four hours in distinct groups of animals after the i.pl. of Bjv (7 µg/100 µL). Animals injected with saline (100 µL/paw) were used as a control group for each evaluated time. Results are expressed as the mean ± SEM of 5–8 animals per group. Analysis performed by two-way ANOVA, followed by Tukey’s test (*p* < 0.05). *** *p* < 0.001, **** *p* < 0.0001 in relation to baseline; # *p* < 0.05, ## *p* < 0.01 in relation to the respective control group (saline).

To characterize Bjv-induced hyperalgesia, animals were injected by intraplantar (i.pl.) route with the venom (7 µg/100 µL) or saline (100 µL) and subjected to the paw pressure test before (baseline (BL)) and 2 or 4 h after the treatments. The results show, at the two evaluated time points, a significant decrease in the nociceptive response to the mechanical stimulus in animals injected with Bjv compared with both baseline and saline groups (Figure 1), characterizing and confirming the hyperalgesic effect of the *Bothrops jararaca* venom.

### 2.2. Spinal Microglia Are Involved in Bjv-Induced Hyperalgesia

**Figure 2:**
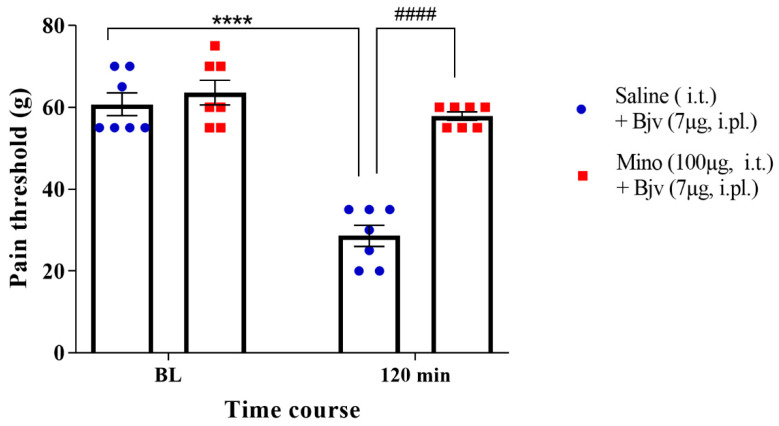
Nociceptive threshold represented in grams (g) of animals evaluated by the paw pressure test. The measures were obtained before the treatments (baseline (BL)) and two hours (120 min) after the i.pl. injection of Bjv (7 µg/100 µL). Minocycline (mino; 100 µg/50 µL, i.t. route) or vehicle (saline; 50 µL, i.t.) were administered immediately after baseline measurement and the Bjv was injected 30 min after these treatments. Results are expressed as the mean ± SEM of 7 animals per group. Analysis performed by two-way ANOVA, followed by Tukey’s test (*p* < 0.05). **** *p* < 0.0001 in relation to baseline; #### *p* < 0.0001 in relation to saline + Bjv group.

In order to explore the potential role of microglia in the hyperalgesic effect induced by Bjv, we treated animals with minocycline intrathecally, 30 min prior to the intraplantar administration of Bjv. The results of our study provide compelling evidence that pretreatment with minocycline led to a remarkable suppression of the decrease in the nociceptive threshold induced by *Bothrops* venom (Mino + Bjv), in contrast with the control group (Sal + Bjv) (Figure 2). This significant difference strongly suggests the involvement of microglia in the hyperalgesic process induced by the Bjv.

### 2.3. Bjv Induces Increased Glial Cells and Neurons Marker Expression

**Figure 3:**
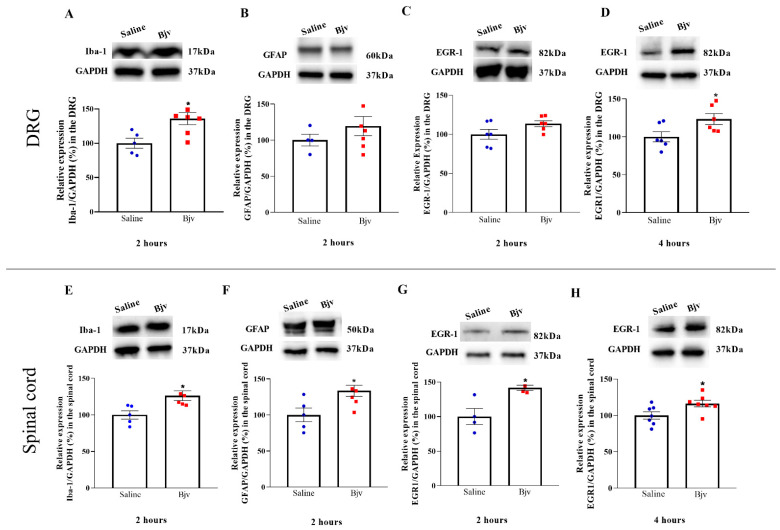
Investigation of glial and neuronal marker expression after Bjv-induced hyperalgesia. After 2 (**A**–**C**,**E**,**F**) or 4 h (**D**,**H**) of saline (100 µL, represented by the blue circle symbol) or Bjv (7 µg/100 µL, represented by the red square symbol) i.pl. injection, rats were subjected to the paw pressure test, sedated, euthanized and the samples of the ipsilateral dorsal root ganglia (DRG, (**A**–**D**)) and lumbar spinal cord (SC, **E**,**F**) collected. The expression of Iba-1 (**A**,**E**), GFAP (**B**,**F**) and EGR-1 (**C**,**D**,**G**,**H**) was evaluated by Western blotting assay. GAPDH was used as the loading control. Results were normalized by defining the control group (saline) as 100%. Results are expressed as the mean ± SEM of 4 to 7 animals per group and were analyzed by the unpaired Student’s *t*-test. * *p* < 0.05 in relation to saline group.

The present study aimed to investigate the participation of the peripheral and central nervous system in the development of Bjv-induced hyperalgesia. To achieve this, Western blotting assays were employed to assess the protein expression of markers for macrophages and microglial cells (Iba-1), satellite cells and astrocytes (GFAP) and the transcription factor expressed in the nucleus of activated neurons (EGR1). Regarding Iba-1, this marker displayed increased expression after 2 h in both the DRG and spinal cord, when comparing the venom and saline groups (Figure 3A,E), coinciding with the peak of Bjv-induced hyperalgesia. In contrast, the GFAP exhibited only a significant increase in the spinal cord at the second hour after venom treatment (Figure 3F), with no changes in the expression between groups in the DRG (Figure 3B). Concerning the marker EGR1, we observed a significant increase in the DRG at 4 h after Bjv treatment (Figure 3D), with similar expression amongst groups at 2 h (3C). Conversely, in the spinal cord, EGR1 expression showed an elevation at both 2 and 4 h after venom injection (Figure 3G,H).

### 2.4. Participation of the Peripheral NK1 Tachykinin Receptors in the Hyperalgesic Effect of Bjv

**Figure 4:**
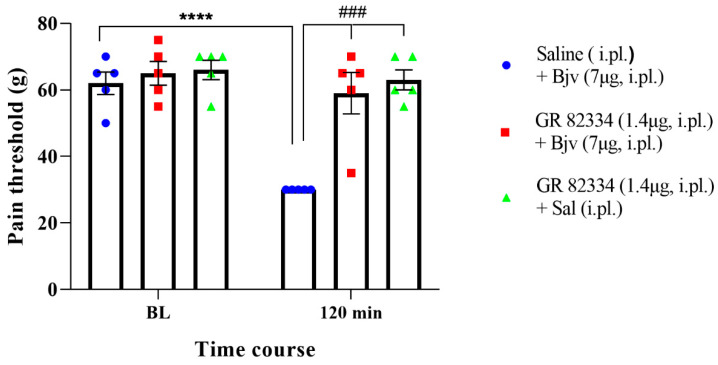
Nociceptive threshold represented in grams (g) of animals evaluated by the paw pressure test. The measures were obtained before the treatments (baseline (BL)) and two hours (120 min) after injection of Bjv or saline. Immediately after baseline measurement, the animals were treated by i.pl. with GR 82334 (1.4 μg/50 μL) or vehicle (saline, 50 µL) and after 15 min the Bjv (7 µg/50 µL, i.pl.) or saline (50 μL, i.pl.) were injected. Results are expressed as the mean ± SEM of 5 animals per group. Analysis performed by two-way ANOVA, followed by Tukey’s test (*p* < 0.05). **** *p* < 0.0001 in relation to baseline; ### *p* = 0.0001 in relation to saline + Bjv group.

To investigate the involvement of peripheral NK1 receptors in Bjv-induced hyperalgesia, we conducted an evaluation by administering the GR 82334 (NK1 receptor antagonist) to the animals by i.pl. route, 15 min prior to the Bjv injection (GR 82334 + Bjv). The compelling results that were obtained demonstrate that the NK1 receptor antagonist completely inhibited the hyperalgesic response induced by Bjv (Figure 4), showcasing its crucial role in mediating this effect. Notably, treatment with GR 82334 before saline did not alter the nociceptive threshold of the animals, confirming the specific action of the NK1 antagonist on the Bjv effect (Figure 4). Additionally, pretreatment with saline did not interfere with venom-induced hyperalgesia (Figure 4).

### 2.5. Expression of NK1 Receptors in the Spinal Cord Is Increased after Bjv-Induced Hyperalgesia

**Figure 5:**
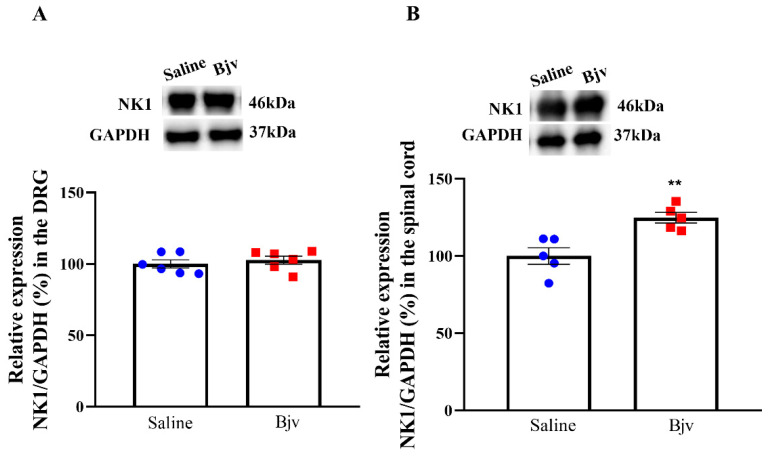
Evaluation of NK1 receptor marker expression after Bjv-induced hyperalgesia. Rats were injected by i.pl. with Bjv (7 µg/100 µL, represented by the red square symbol) or saline (100 µL, represented by the blue circle symbol) and after 2 h submitted to the paw pressure test, sedated, euthanized and samples from the L4–L6 region of the ipsilateral side of the dorsal root ganglia (DRG, **A**) and lumbar spinal cord (SC, **B**) collected. The expression analysis was performed by Western blotting assay. GAPDH was used as the loading control. Results were normalized by defining the control group (saline) as 100%. Results are expressed as the mean ± SEM of 5 to 6 animals per group and were analyzed by the unpaired Student’s *t*-test. ** *p* < 0.01 represents a significant difference compared with the control group (saline).

In order to examine NK1 tachykinin receptor expression in the nervous system, we performed analyses on samples obtained from the DRG and spinal cord of animals injected with either saline or Bjv and evaluated in nociceptive test. The results of our study reveal an increase in the expression of NK1 receptors in the spinal cord of the Bjv-injected group compared with the saline control group (Figure 5B). This finding suggests a potential upregulation of NK1 receptors in the spinal cord as a response to Bjv-induced hyperalgesia. However, no such increase in receptor expression was observed in the DRG (Figure 5A), indicating that the venom induces a differential pattern of NK1 receptor modulation between the peripheral and central nervous systems.

## 3. Discussion

Envenomation resulting from *Bothrops* genus snakebites represents a significant public health concern [32]. One notable aspect of envenomation by these snakes is the occurrence of intense pain and hyperalgesia at the bite site [1,5]. An understanding of the cellular and molecular mechanisms underlying the pain induced by venomous snake bites, such as those of *Bothrops jararaca* (Bjv), is of paramount importance for developing effective therapeutic strategies. While the inflammatory mediation of pain caused by snakebite has been extensively studied, limited knowledge exists regarding the mechanisms involving the nervous system in the hyperalgesic effect induced by Bjv. In this study, we sought to characterize the involvement of specific cell types in the nervous system and the role of NK1 receptors in Bjv-induced hyperalgesia. Our findings reveal that the hyperalgesia induced by Bjv is associated with the activity of spinal cord microglial cells and peripheral NK1 receptors. Additionally, we noted an upregulation in the expression of markers of microglial and astrocytic cells, neurons, and NK1 receptors, indicating that the mechanisms governing pain transmission in Bjv-induced hyperalgesia are related to the increased activity of these components present in the spinal cord.

Initially, we conducted experiments to evaluate the effect of intraplantar injection of Bjv in animals submitted to the paw pressure test. The results reveal a decrease in the nociceptive threshold at 2 and 4 h after venom injection, as previously demonstrated by our group [17] and confirming the hyperalgesic effect of Bjv. This observation is consistent with previous studies, supporting the notion that Bjv induces pain hypersensitivity [9,14,17]. In this context, the involvement of substances present in the venom, along with inflammatory mediators released by immune cells, in mediating this effect has been widely evaluated, particularly in the first hours after administration of Bjv [1,5,6,8,9,14,16,17]. Therefore, understanding the aspects related to the activation of nociceptive response transmission pathways in the nervous system during hyperalgesia underscores the importance of exploring the cellular and molecular mechanisms at play. To delve deeper into this, we selected a 2 h time period for the subsequent assessments, focusing on an earlier stage of the process. This timeframe may be crucial for understanding immediate changes in neurons and glial cells within the nervous system, as well as the potential activation of NK1 receptors, which contribute to the transmission of pain signals [22,23,31,33]. Furthermore, in clinical and therapeutic terms, understanding and controlling the mechanisms involved in the pathophysiology of pain induced by Bjv at the onset of the process can assist in the development of strategies with which to prevent pain persistence, as poorly managed inflammatory pain is one of the factors that can lead to the chronification of the process [34].

To assess the contribution of spinal cord microglial cells in the hyperalgesic response to Bjv, we employed minocycline, an inhibitor of microglial activation [35,36]. Notably, pretreatment with intrathecal administration of minocycline led to a significant suppression of the hyperalgesic response induced by Bjv. These data strongly implicate the involvement of spinal microglia in the venom-induced hyperalgesic process, highlighting their potential role in amplifying this phenomenon. These findings are reinforced by a study conducted by Ferraz et al. [12], where they observed that jararaghin, one of the components of Bjv, induces hyperalgesia by mechanisms involving the activation of microglia in the spinal cord. Moreover, evidence of microglial activation in the spinal cord has been reported during acute inflammatory pain across various hyperalgesia models, such as the hyperalgesia induced by complete Freund’s adjuvant (CFA) [37], zymosan [38], formalin [22] carrageenan [39], and two components of *Bothrops asper* snake venom, Lys49 and Asp49 secretory phospholipase A_2_ (sPLA_2_) [19]. The participation of microglial cells in the early stages of this process is supported by previous evidence, as their activation was determined 4 h after CFA or 1–2 h after formalin injection [22,37]. Similarly, our results also demonstrate the involvement of microglial cells in the initial stage of the hyperalgesic effect induced by Bjv.

In order to elucidate the role of different cell types present in the DRG and lumbar spinal cord in Bjv-induced hyperalgesia, Western blot assays were conducted to evaluate the protein expression of specific markers in samples obtained after paw pressure tests in animals injected with the venom.

Firstly, we examined the expression of EGR1, a transcription factor commonly utilized to delineate the activation neuronal [40,41]. Our results demonstrate the increased expression of the neuronal marker EGR1 in the spinal cord at both of the time points evaluated following Bjv injection. On the other hand, a significant increase in EGR-1 expression in the DRG was detected only four hours after hyperalgesia induction. These data suggest greater activity of neurons in transmitting nociceptive stimulus from the periphery to the central nervous system after Bjv-induced hyperalgesia; however, the neuronal response to the stimulus appears to occur differently between the CNS and PNS, as in the spinal cord, this response is already observed in the early stages of the process, whereas this effect is triggered later in the DRG.

EGR1 plays a pivotal role in synaptic plasticity and nociceptive signaling through pathways such as the MAP-kinase pathway [42,43]. Furthermore, Ko et al. [44] have reported that knockout animals lacking EGR1 exhibited a reduced pain response to formalin injections compared with wild-type animals, reinforcing the intricate involvement of neuronal factors in amplifying the sensation of pain. Numerous studies have demonstrated that EGR-1 is expressed in neurons of the spinal cord following noxious stimulation [45,46,47]. Moreover, the expression of EGR-1 in the dorsal horn of the spinal cord can be detected early after hyperalgesia induction, as demonstrated by Dale et al. [48]. Our data are supported by the findings in the literature, as we have also observed an increase in the expression of EGR-1 in the spinal cord during the early stages of Bjv-induced hyperalgesia, which may be associated with greater activity of neurons in this region of the central nervous system.

The information regarding the expression of EGR-1 by neurons of the DRG is still limited. It has been shown that, in DRG cultures enriched with sensory neurons, there is an increase in EGR-1 mRNA in response to treatment with nerve growth factor (NGF) [49]. Additionally, the expression of EGR-1 was observed in DRG cells from naive rats, and approximately half of the nociceptors were found to be EGR-1 positive [50]. In this way, the results shown here provide new insights into the expression of EGR-1 in the DRG after a nociceptive stimulus, and raise the hypothesis that sensory neurons present in the DRG are also more active during Bjv-induced hyperalgesia.

In order to complement the findings regarding the involvement of microglia in the hyperalgesic effect produced by Bjv, the expression of the marker Iba-1 was assessed. This marker is commonly used to identify and characterize microglial cells and macrophages, and its expression may be increased during inflammatory processes or immune responses in injured tissues, as well as in response to painful stimuli [22,51,52,53]. The expression of Iba-1 was increased in the lumbar spinal cord at the onset of the Bjv-induced hyperalgesia, reinforcing the involvement of microglia in this effect, as indicated by the results obtained from the treatment of animals with minocycline. Studies conducted by Gu and colleagues [22] have demonstrated that microglia not only mediate the acute inflammatory pain induced by formalin but also contribute to the increased neuronal activity in the dorsal horn of the spinal cord. Our findings corroborate those obtained in this study, as we also demonstrated an increase in neuronal and microglial activity during the initial phase of hyperalgesia induced by Bjv, similar to the timeframe observed by these authors. In this sense, it is plausible to speculate that the heightened neuronal activity observed here, measured by EGR1 expression in the spinal cord, may be correlated with the activation of spinal microglial cells. However, additional investigations are necessary to confirm this hypothesis. Our data also reveal an increase in Iba-1 levels in the samples obtained from the DRG. Given that microglial cells are typically present only in the CNS [54], the increased expression of this marker in the DRG during hyperalgesia induced by Bjv is likely associated with macrophage activity. In sensory ganglia, two distinct populations of macrophages can be identified following an inflammatory stimulus or nerve injury, the resident macrophages associated with neurons and the monocyte-derived macrophages that infiltrate the ganglia [55,56,57,58,59]. As the WB technique only detects the increase in Iba-1 expression, it is not possible to determine which type of macrophage population present in the DRG is involved in the effect triggered by Bjv.

Furthermore, the analysis of GFAP expression, utilized as an activity marker for astrocytes in the CNS and satellite cells in the PNS [60], revealed a significant increase only in the spinal cord in the hyperalgesia caused by Bjv. Previous studies using toxins isolated from *Bothrops asper*, specifically the phospholipases Asp-49 and Lys-49, have shown their capacity to induce hyperalgesia and increase GFAP labeling in the spinal cord of rats [19]. Additionally, Ferraz et al. [12] have reported this same effect with Jararaghin, a metalloprotease isolated from Bjv. The increased expression of GFAP was associated with augmented activity of astrocyte cells triggered by these toxins [12,19]. In this way, the data presented here also support the idea that astrocytes present in the spinal cord may be more active at the onset of venom jararaca-induced hyperalgesia. As for satellite glial cells (SGCs), studies have demonstrated elevated levels of GFAP labeling in sensory ganglia after inflammatory, pathological, and/or chronic pain [60,61,62]. However, we did not observe any alteration in the expression of GFAP in the DRG after the injection of Bjv. Some studies have reported that, even during inflammatory pain, the increase in GFAP in sensory ganglia is observed in the later phase of the painful process [63,64]. This suggests that SGCs may play a more significant role in modulating pain in chronic and long-term pathologies.

The lack of an observed increase in GFAP in the DRG during the second hour after Bjv-induced hyperalgesia suggests that SGCs are not initially involved in this process. Conversely, our results indicate that astrocytes may contribute to the early mechanisms involved in processing the painful stimulus caused by Bjv.

Our study also explored the role of NK1 receptors in Bjv-induced hyperalgesia. The intraplantar administration of the NK1 receptor antagonist GR 82334 prior to Bjv injection completely inhibited the hyperalgesic response induced by the venom, indicating the crucial role of local NK1 receptors in mediating this effect. As previously mentioned, NK1Rs are involved in pain transmission and modulate inflammatory responses, and in peripheral tissues are found in lymphocytes, monocytes, and macrophages [29]. Activated NK1Rs can regulate the expression of cytokines and chemokines, as well as the activation of nuclear factor-kappa-b (NF-kB), resulting in increased synthesis of pro-inflammatory mediators that contribute to the development of hyperalgesia, such as nitric oxide and prostaglandins [65,66]. Previous reports have shown that Bjv induces hyperalgesia through prostaglandin-dependent mechanisms [14], and also leads to increased nitric oxide production by macrophages [67]. Therefore, it is plausible that blocking the receptors of NK1 with its antagonist would inhibit the synthesis of these inflammatory mediators that are essential for hyperalgesia induction. Furthermore, our results provide additional insights regarding the involvement of neurogenic factors in local reactions caused by Bjv, given that Gonçalves and Mariano [25] have suggested that local hemorrhage induced by this venom is related to neurogenic inflammation, and that we have demonstrated that blocking NK1R, which is primarily activated by the neuropeptide substance P, plays a key role in the genesis of neurogenic inflammation and pain [29] and completely inhibits the hyperalgesia triggered by Bjv. In addition to the peripheral involvement of NK1Rs, we also evaluated the expression of these receptors in samples obtained from the DRG and spinal cord, as NK-1 receptors have been detected in neurons present in these tissues [68,69]. We observed that only in the spinal cord was an increase in expression of the specific marker for NK1 receptors detected following hyperalgesia induced by Bjv, while in the DRG no difference was obtained. These data indicate that the venom induces a differential pattern of NK1 receptor modulation between the peripheral and central nervous system. The NK1 receptors and their main ligand (SP) have been strongly linked in the mediation of nociception, and a variety of noxious stimuli that evoke the release of SP can activate NK1 receptors in neurons located in lamina I of the dorsal horn of the spinal cord [70]. In this sense, the inflammatory process associated with tissue injury triggers the increase of expression of SP and the NK1Rs in the spinal cord [70]. In a study using two distinct inflammatory agents to induce nociception, formalin or complete Freund’s adjuvant (CFA), it was demonstrated that intraplantar injection of these irritants caused increased NK1R mRNA expression in the right (ipsilateral) lumbar dorsal horn [71]. The results presented here align with those demonstrated by Krause and colleagues [71], as we also observed an increase in NK1R expression in the lumbar region of the spinal cord on the ipsilateral side following intraplantar injection of Bjv. However, due to the technique used to detect the marker expression for this receptor, we cannot definitively state whether the increase occurred in lamina I of the dorsal horn of the spinal cord. Nevertheless, a hypothesis worth considering is that increased expression of NK1Rs may occur in both neurons and glial cells within the spinal cord, as previous studies have shown that microglial and astrocytic cells express these receptors [30,72,73]. Regardless of which cell is expressing the NK1 receptor, the data presented suggest that hyperalgesia induced by Bjv may stimulate the expression of this receptor in the central nervous system, possibly through mechanisms related to the action of SP released by primary sensory neurons. Future studies will be conducted to clarify the role of SP/NK1R interaction in mediating peripheral and central hyperalgesia evoked by *Bothrops jararaca* venom.

## 4. Conclusions

In conclusion, our study provides valuable insights into the cellular and molecular mechanisms underlying pain induced by *Bothrops jararaca* venom. The findings of the involvement of microglia, astrocytes, and NK1 receptors in Bjv-induced hyperalgesia contribute to a better understanding of the mechanisms involved in this symptom, which may aid in the development of more effective and targeted therapeutic interventions to alleviate the pain caused by snakebite envenomation.

## 5. Materials and Methods

### 5.1. Animals

The experiments were performed on male Wistar rats, weighing 170–190 g, supplied by Butantan Institute Central Animal Facility. Animals were housed in groups of 5 per cage, kept with free access to food and water, and maintained in an appropriate sound-proof room under a controlled light cycle (12 h/12 h dark/light cycle) and temperature (22 ± 2 °C). All procedures were performed according to the guidelines for the ethical use of conscious animals in pain research, following the International Association for Study of Pain (IASP) [74] and approved by the Institutional Animal Care Committee of the Butantan Institute (CEUAIB, protocol number 127006519). The Animal Research Reporting of In Vivo Experiments (ARRIVE) guidelines were followed while conducting the study.

### 5.2. Experimental Design

**Figure 6:**
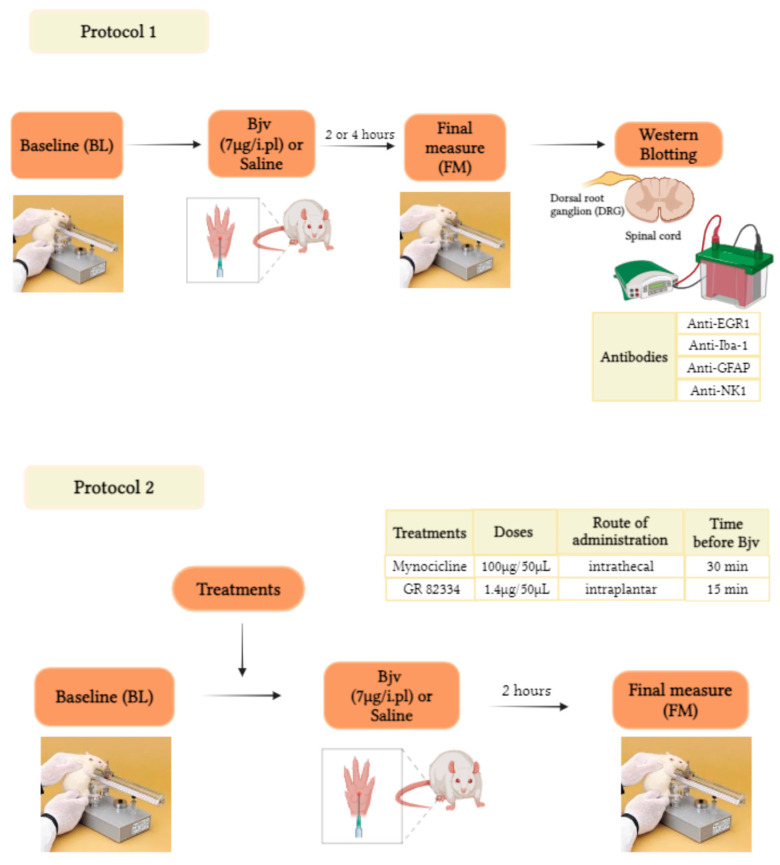
Experimental design.

The experiments were performed according to Figure 6. The number of animals used in each group ranged from 4 to 8, depending on the experiment, as shown in the figures and described in the legends. To characterize the involvement of specific cell types in the nervous system in Bjv-induced hyperalgesia, the animals’ pain sensitivity was initially evaluated before (baseline (BL)) and 2 or 4 h after intraplantar (i.pl.) injection of Bjv (7 µg/100 µL) or saline (100 µL) in different groups of animals. This test aimed to assess the hyperalgesic effect of Bjv and to induce the activation of pain transmission pathways in the nervous system following the application of a mechanical stimulus. Immediately after the evaluations, rats were sedated, euthanized, and samples were collected from the ipsilateral side of the dorsal root ganglia (DRG) (a pool of 3 DRGs corresponding to L4–L6) and the lumbar segment of the spinal cord (SC) (corresponding to L4–L6, SC) for Western blotting assays. These assays were conducted to further analyze protein expression related to different cellular markers (Protocol 1). In Protocol 2, following the baseline (BL) assessment, animals received intrathecal (i.t.) treatment with minocycline (100 µg/50 µL) or saline (50 µL) 30 min before i.pl. injection of Bjv, or with GR 82334 (1.4 μg/50 μL, i.pl.) or saline (50 µL, i.pl) 15 min prior to venom or saline administration. Pain sensitivity in the animals was reassessed (final measure (FM)) 2 h after the injection of Bjv or saline.

### 5.3. Venom

A pool of lyophilized venom from adult specimens of *Bothrops jararaca* snakes was obtained from the Laboratory of Herpetology, Butantan Institute, Brazil. Venom was weighed at the moment of use and dissolved in sterile saline (7 µg/100 µL). Animals were injected by intraplantar (i.pl) route with either 100 µL of sterile saline solution (control group) or 100 µL of *Bothrops jararaca* venom (Bjv, 7 µg/paw). This dose of venom was determined based on a previous study, where we observed that Bjv induces hyperalgesia for up to 4 h following intraplantar injection in rats evaluated in the paw pressure test [17].

### 5.4. Evaluation of Mechanical Hyperalgesia

To evaluate mechanical pain threshold, we applied the rat paw pressure test [75], using an analgesy-meter (Ugo Basile, Gemonio, Varese, Italy), In this test, an increasing pressure was continuously (16 g/s) applied to the hind paw of the rats and interrupted when the animals reacted by withdrawing the paw. The force needed to induce this behavior was recorded and interpreted as pain threshold. To reduce stress, one day before the experiment, animals were habituated to the testing procedure and the apparatus. On the day of the experiment, mechanical hyperalgesia was evaluated before (baseline (BL)) and 2 h or 4 h after i.pl. injection of the Bjv or saline (final measure).

### 5.5. Drugs

Minocycline (Sigma-Aldrich, Merck KGaA, Darmstadt, Germany), a pleiotropic broad-spectrum tetracycline antibiotic, widely used to inhibit microglial activation and as a specific microglial cell inhibitor, was dissolved in saline (100 µg/50 µL) and given intrathecally (i.t.) 30 min before Bjv (7 µg/100 µL, i.pl.). The control group was injected with saline (50 µL, i.t.) prior to the venom. The dose of minocycline used and the intrathecal injection were determined as previously described [19,36].

GR 82334 (Sigma-Aldrich, Merck KGaA, Darmstadt, Germany), a potent and specific reversible tachykinin NK1 receptor antagonist, was dissolved in saline (1.4 µg/50 µL) and given by the intraplantar route (i.pl) 15 min before the Bjv (7 µg/50 µL, i.pl.) or saline (50 µL, i.pl.) [76]. The animals injected with saline prior to venom were also evaluated. The dose of GR 82334 used was based on a study conducted by Zanchet et al. [76].

### 5.6. DRG and Spinal Cord Samples

After evaluating the paw pressure test (Section 2.1), rats were deeply anesthetized with isoflurane and euthanized by a section of carotid arteries and jugular veins, followed by dissection in the dorsal media area in the L4–L6 segments and collection of the dorsal root ganglion (DRG) and spinal cord (SC). The samples were obtained from the ipsilateral side of the DRG (a pool of 3 DRGs corresponding with L4–L6) and lumbar segment of the SC (L4–L6) and analyzed by Western blot.

### 5.7. Western Blot

The samples collected were homogenized in RIPA protein lysis buffer (50 µL for DRG and 150 µL for SC) containing cocktail protease inhibitor and phosphatase 1:300 (Sigma-Aldrich, Merck KGaA, Darmstadt, Germany) and centrifuged at 15,000× *g* for 20 min at 4 °C. The total protein concentration of the supernatant was determined using the BCA protein detection kit (Novagen, Merck Millipore, Merck KGaA, Darmstadt, Germany, #71285). Aliquots containing 20 µg (DRG) or 30 µg (SC) of protein were boiled in Laemmli buffer for 7 min. Then, proteins were separated on polyacrylamide gel electrophoresis SDS PAGE (8, 12 or 4–20%, according to the protein to be detected) and transferred to nitrocellulose membranes (Bio-Rad Laboratories, Hercules, CA, USA, #1620115). The membranes were blocked for 60 min with 5% bovine serum albumin (BSA) in TBST (20 mM Tris-HCl, 150 mM NaCl, and 0.1% Tween 20). Subsequently, membranes were incubated with antibodies anti-ionized calcium-Binding adapter molecule 1 (anti-Iba-1) (1:1000, Abcam, EPR16588, Cambridge, MA, USA, #ab178846), anti-glial fibrillary acid protein (anti-GFAP) (1:5000, Sigma-Aldrich, Merck KGaA, Darmstadt, Germany, #G3893), anti-early growth response protein 1 (anti-EGR1) (1:250, Santa Cruz Biotechnology Inc., Dallas, TX, USA, #SC-189) and anti-Neurokinin 1 (anti-NK1, 0.125 µg/mL, Abcam, Cambridge, MA, USA, #ab131091) overnight at 4 °C. After washing with TBS-T, membranes were incubated with the peroxidase-conjugated secondary antibodies, as anti-rabbit (1:5000, Abcam, Cambridge, MA, USA, #6721-1) for Iba-1, EGR-1, NK1 and GAPDH (glyceraldehyde-3-phosphate dehydrogenase) or anti-mouse (1:5000, Abcam, Cambridge, MA, USA, #205719) for GFAP, at room temperature for 120 min. The enhanced chemiluminescence (ECL Kit, Thermo Fisher Scientific, #34076) with a digital image captured system (UVITEC, Cambridge, UK) was used to detect the bands. The optical densitometries of the bands were quantified using the ImageJ 1.53k software (National Institutes of Health, Bethesda, MD, USA). Protein equivalence was performed with anti-GAPDH (1:20,000, Abcam, Cambridge, MA, USA, #ab9485) used as the loading control. Results were normalized by defining the control group (saline) as 100%.

### 5.8. Statistical Analyses

All data are presented as means ± SEM in each group per experiment. Results of Figure 1, Figure 2 and Figure 4 were analyzed using two-way repeated measure analysis of variance (ANOVA) followed by Tukey’s post hoc test. Student’s *t*-test was performed to analyze Western blot data (Figure 3A–H and Figure 5A,B). GraphPad Prism 8.0 (GraphPad Software Inc., Boston, MA, USA) was used for analysis of the data and statistical significance was considered as *p* < 0.05.

## Data Availability

The original contributions presented in the study are included in the article. Further inquiries can be directed to the corresponding author.
